# Addressing the mental health needs of victims-survivors of technology-assisted child sexual abuse and grooming

**DOI:** 10.3389/fpsyg.2026.1642503

**Published:** 2026-05-20

**Authors:** Karen Garland, Lawrence Jordan, Victoria Green

**Affiliations:** Marie Collins Foundation, Northallerton, United Kingdom

**Keywords:** impact, mental health, needs, TACSA, victims-survivors

## Abstract

Direct work with victims-survivors of technology-assisted child sexual abuse who experienced online grooming reveals a constellation of mental health and support impacts, flowing from the abusive experience. This practitioner report highlights the areas of mental load affecting victims-survivors as a direct result of grooming. Juggling these psychological burdens produces lingering mental health impacts for victims-survivors. The report further outlines priorities for support services to target when working with victims-survivors, to assist in dismantling the detrimental psychological harms inflicted due to grooming. The cognitive pressure which TACSA victims-survivors experience includes feelings of culpability due to their self-reported inability to say no to their abuser or remove themselves from the online environment. This provokes an onward sense of responsibility for their own abuse, which is compounded by guilt due to how their bodies responded to what happened. The existence of images of their abuse forces silence upon victims-survivors, due to shame and embarrassment. Silence is also an attempt by victims-survivors to pre-emptively alleviate any potential societal victim-blaming. Victims-survivors express concern that abuse images potentially serve as evidence of their involvement and compliance with the grooming and abuse, inviting blame apportionment. Practitioners routinely need to unpack and gently challenge the mental distortions that victims-survivors express. Specifically, these include empowerment messages around lack of victim fault for their abuse, placing responsibility solely with the offender, normalizing physiological and psychological responses to abuse to counteract feelings that their minds and bodies welcomed the abuse. Practitioners can offer a unique view of victim-survivor experiences of mental health struggles following grooming, alongside the necessary supportive approaches to aid recovery.

## 1 Introduction

The Marie Collins Foundation (MCF) is a UK charity uniquely focused on addressing and responding to the recovery needs of victims of technology-assisted child sexual abuse (TACSA). Direct and indirect support work with victims of TACSA is a primary strand of MCF’s field of engagement and advocacy strategy. The promotion of lived experience of victims and survivors is central to MCF, through both our direct work with victims and their families and the expertise of our Lived Experience Group of adult survivors. Learning from direct work is used to promote a victim-focused recovery approach and to influence improved prevention and response to TACSA, both domestically and internationally. We also create and deliver specialist training to professionals, including police and social workers, across the globe to equip them for better intervention and support for those who have experienced TACSA and to promote a victim focused, recovery approach.

TACSA refers to a range of predatory and abusive actions whereby technology is a key component of the abuse occurrence (Quayle et al., 2023; Hamilton-Giachritsis et al., 2020). This may include sexual conversations with children, online grooming, coercively produced sexual images or videos, financially motivated sexual exploitation or a combination of such abuses. Though technology is the medium for abuse, this does not necessarily mean the abuse is confined online. Indeed, while it may occur solely in that domain, it may also begin online and become transmitted to an offline experience at some point. The abuse can traverse online and offline spaces (Hanson, 2017b).

Crucially, the wider research on gender-based violence has challenged the view that impacts from online abuse are less serious than contact forms of abuse and violence (Whittle et al., 2013; Huber, 2023). The research base has revealed that the effects of TACSA on victims-survivors are equal and comparable with victims-survivors of offline-focused CSA (Joleby et al., 2020; Hamilton-Giachritsis et al., 2020; Jonsson et al., 2019). Mixed methods research by Hamilton-Giachritsis et al. (2020) is particularly helpful in demonstrating how TACSA impacts are on a par with abuse occurring offline. This study also detects important ways that technology assists the abuse and the unique manifestation of the impact of TACSA, which includes the circulation of sexual imagery *ad infinitum* and corresponding opportunities for continuing revictimization. This is a significant difference from offline CSA abuse and impacts, which are typically confined to instances of contact abuse occurrence only. Those who experience online forms of CSA appear to be at an increased risk of experiencing grave mental health impacts (Say et al., 2015). Furthermore, given that TACSA has the potential for additional sharing, downloading and even increasing alteration via AI-generators [INHOPE Annual Report (INHOPE, 2025)], victim impact and recovery is a very complicated and non-linear journey (Hanson, 2017b). Indeed, offenders appear to deliberately harness technology to control victims through the threat of dissemination (Say et al., 2015). Unfortunately, wider public awareness and discourse is not entirely in-step with this academic evidence base. This is also reflected within the sentencing tariffs for offline CSA versus TACSA across the United Kingdom. TACSA offenses generally command much lower sentences than contact CSA offenses, with a high number of suspended sentences deployed for those convicted of TACSA offenses (Centre of expertise on child sexual abuse, 2025). This tends to suggest that the effects of TACSA are not well understood and that the resultant harm is considered less serious than other forms of CSA.

The unique impact of TACSA will produce specific therapeutic needs for victims. Although victimization which occurs via the medium of technology may produce corporeal reactions to the harm, this practitioner report will focus on the mental health impacts only. It is important that counselors and those providing therapeutic care and support understand the manifestation of TACSA effects on the mental load of victims. This may include phenomena such as fear and hypervigilance, dissociation, misplaced self-blame, with corresponding experiences of shame. Without an accurate appreciation for the psychological imprint of the TACSA experience, the therapeutic alliance could be compromised, and the effect of mental health support could be ineffectual.

This report draws on MCF’s direct experience of engaging with victims and survivors of various forms of TACSA forming a composite view of the mental health effects and corresponding needs. Our responses refer to direct work with child victims and their parents, or direct parental support. The majority of children were female, with a concentration in ages 11–15. Our direct work is not therapeutic counseling. Rather, we provide tailored, person-centered support and advocacy. We meet with the parent and/or child, listening to their experiences, providing empathic and structured guidance for them on the nature of TACSA. We also outline legal and policy issues and rights relevant to their circumstances, as well as offering first-hand support in any key meetings with statutory agencies (including police, social work and education). We may also provide debriefing of meetings, where requested. Our level of supportive involvement is determined by the child and parent. It can include both in-person, online or phone calls, (or a combination of these). Some families seek minimal with MCF (2–3 sessions), while other families request extensive and ongoing support work. This can depend on the particulars of the TACSA experience and criminal justice processes, in particular. Overall, we work to improve responses, strengthen recovery, and ensure that victims and survivors receive the support they need and deserve.

The analysis is derived from a desktop review of our direct work cases files. Some anonymous examples will be offered from the children MCF have supported in the selected period (2022–2025). In completion of this practitioner report, data was only used where (1) it would not identify the family or those associated with victim-survivors and (2) consent had been obtained in accordance with MCF GDPR processes and through verbal discussion between MCF practitioners and the family. MCF has identified critical areas of focus for clinicians working therapeutically with victims. The report suggests four key areas where counselors should direct their support, to unpack the mental burdens which victims and survivors experience, offering the greatest areas for potential growth and recovery following TACSA.

## 2 Composite mental health effects

Between 2022 to 2025, MCF engaged in forty-three responses to a range of sexual abuse and harm that occurred online or through the use of technology. This report draws on all of these responses, which included experience of online grooming in twenty-five (58.1%) of these referrals to our services. Recurring themes emerge when working with those who have experienced online grooming and TACSA, pivoting around a primary area which encompasses the severe mental health impact experienced by victims stemming directly from their abusive experience. These mental health effects will be discussed hereafter as a composite of MCF practitioner experience with victims of TACSA. Some anonymous examples will be offered for those MCF have supported in the selected period.

## 3 Culpability, complicity, responsibility

Victims frequently spoke to practitioners within MCF about their feelings of distress originating from their perception that they failed to stop the abuse from happening. Despite the limitations due to their age and developmental stage at the time the TACSA occurred, they expressed a sense of frustration and self-directed anger for not disrupting the abuse. Given the abuse took place online, many felt they should have been able to extricate themselves from the online environment, to protect themselves. This is despite some not realizing at the time that what they were actually experiencing was sexual abuse.

Victims also expressed confusion about the role they played in the abuse, the conversations they contributed to and the activities they engaged in. The initial engagement was often noted as rewarding and exciting, with someone talking an interest in them, talking about things they could not talk to others about, including engaging in sexual talk. This made them feel as if they were “willing” participants. Victims expressed shame over someone else seeing these interactions and potentially judging them.

This self-reported “failure to say no” to the TACSA resulted in intense feelings of culpability, complicity and responsibility. During the initial support sessions, victims were unable to comprehend how their age and capacity validly contributed to their lack of agency in avoiding or stopping the abuse. Furthermore, there was an initial lack of insight into how the perpetrator utilized various strategies to secure the victim’s compliance, to avoid victim resistance to the abuse and to ensure it stayed a secret. Perpetrator techniques appear to be highly effective in securing victim compliance, but the sophistication of these behaviors also produced a particularly pernicious effect on victims, where compliance is mentally metabolized as complicity and responsibility for what happened to them.

## 4 Guilt, shame, embarrassment

Victims reported strong feelings of guilt because of their corporeal response to the abuse. This sense of bodily betrayal was derived from some of the normative physical responses to sexual stimuli. Noting their experiences of arousal, victims expressed a crippling sense of guilt due to the apparent conflict between the harm of being abused with a generally welcomed feeling of sexual arousal and excitement. This contradiction was deeply uncomfortable for them, causing a guilt-ridden paralysis in expressing their experience and the validity of seeking therapeutic help.

In addition, twin feelings of shame and embarrassment were routinely expressed and globally felt by this cohort. These psychosocial impacts were intrinsically connected to the ambiguity over the location of and access to the images of their abuse. Victims reported extreme distress over the whereabouts of the pictures and/or videos of their abuse. Many were concerned about who may have access to the images currently or in the future. Acute concern was often discussed about the potential for friends and family to see or stumble upon the archive of their abuse online. Furthermore, there was considerable anxiety when out in public that they may be recognized from pictures or videos. This was often exacerbated every time media reported someone for viewing and/or downloading Child Sexual Abuse Material (CSAM), leaving victims wondering if their images were among them. Shame and embarrassment responses caused victims and survivors to become insular, mirroring an agoraphobic-type response, to manage and mitigate their worry about who might see their abuse.

## 5 Silence

Building upon the sense of complicity, victims and survivors commented on their reflexive or decisive response of silence. When explored in direct work with MCF, this reaction was expressed as a method to counteract anticipation of being blamed for their abuse, or their expressed sense of culpability. Fear of blame and the consequences of disclosure appeared to motivate a choice of silence. Silence occurred at the point of abuse, but was also in response to TACSA, as a coping mechanism, to avoid experiencing real or imagined objective blame. In some cases practitioners’ reluctance to provide the opportunity for the victim to discuss their abuse resulted in what amounts to tacit collusion with the victim’s silence. Silence resulted in non-intervention, whether this was interpersonal support from trusted allies or formal support and intervention from statutory services. For some TACSA victims, their abuse occurred alongside their siblings, which has resulted in them not coming forward for fear of the siblings being contacted, and the wider family finding out. Silence in this form therefore resembles protection for self and others.

## 6 Blame

Victims and survivors expressed both self-blame and anticipated societal blame. This was closely connected to the documentary evidence of their abusive experience. Victims and survivors were extremely concerned that the pictures and videos, which they had no control over the whereabouts or proliferation of, would serve as an instrument for blame. They painfully and frequently discussed their worries that the depictions of their experience would be met with reproach and negative judgment, regardless of who saw them. They expressed shame and embarrassment about the activities and conversations they engaged in. This extended to the performance of sexual acts and sexual conversations. One victim said “*I felt like I encouraged it when the chat got more sexual. I hated that I liked it.*”

Many victims have reported to MCF that it is not what was done to them, or they were made to do to themselves that has caused the greatest impact. What has affected many victims significantly is the feeling they were duped, conned and used.

“*It felt ok, I liked it at the time, I was not a naïve young kid.*”

This emasculated victims and survivors from seeking timely help and support, believing the images would render them as unworthy victims, unentitled to formal or informal help.

## 7 Composite mental health needs

Direct work with victims and survivors has led the Marie Collins Foundation to identify four important areas which are essential for those delivering therapeutic support to address and consolidate in the clinical setting with victims and survivors of TACSA. These are (1) Validation (2) Empowerment, (3) Apportionment of Blame, and (4) Normalization. If dealt with sensitively by experienced and informed clinicians, these realms can be targeted for deep clinical work which will specifically dismantle the four primary arenas of mental stress already outlined in this report, which victims routinely experience.

## 8 Validation

MCF has frequently observed how important it was to victims to have their experiences heard, believed and empathized with. Grooming techniques and a child’s limited sexual experience can be confusing for the victim, who may feel harmed by their abusive experience but not able to quantify these feelings. When victims have been disbelieved or had their credibility questioned, including by police, and children’s social care they report to MCF that this left them struggling mentally. Instead, when victims have been given time and space to explain their experiences and have been offered onward support to seek justice and healing, they have expressed gratitude and relief in having their abusive experience acknowledged and validated by experts who understand the mechanics of TACSA.

Non-validating responses from professionals in the aftermath of TACSA were reported to or observed by MCF. This has included responses from police forces and social services. The demands of any intervention or investigation needs to be counterbalanced by professionals through maintaining the child’s centrality in the management of the case. This includes awareness and sensitivity to a child’s idiosyncratic reaction to the harm suffered because of their experience.

Statutory services prioritizing the process, rather than the victim’s recovery can negatively compound the mental health impacts on a victim. One victim’s mother described that despite an evident decline in her son’s mental health, dismissive comments were routinely made about his health in comparison to the importance the police had placed on the investigation process.

Lack of respect from practitioners, including police, to the abusive images was highlighted in several of the cases where the victim specifically expressed further distress caused by the management of their image. The recordings are often not seen by professionals as a crime scene, and consideration as to their handling and storage is often not thought about. Families routinely describe that professionals have looked at the images, and one mother supported by MCF still refers to the look of fear on her daughter’s face when the school safeguarding lead invited her to view the pictures of her daughter’s abuse.

This lack of sensitivity to the image appears to detach the child from the evidence of their abuse, creating an impression for the victim that the captured image is of greater importance to the practitioner than the child and their wellbeing.

The framework in which statutory services operate can also deny the victim the validation of what happened. The Criminal Justice System is still criticized for its inhospitable conditions for victims of sexual offenses, with calls for this to be urgently improved upon (Victims’ Commissioner, 2024). A very recent ruling by the Court of Appeal finally overturned a previous ruling that prevented people groomed online and sexually abused for being able to claim criminal injury compensation (Morgan, 2023; Stark, 2007) reminds us that to comprehensively understand harm, we must not only look at what has been done to the victim but equally what is the harm preventing them from doing in the future. Validation of their experience by experienced professionals is a core component in helping victims work toward a positive future. This should include those performing therapeutic clinical work, but also those in the victim’s immediate orbit, who are part of intervention and response processes, such as police and social workers.

## 9 Empowerment

At its most basic, abuse is the imbalance of power one over another. The perpetrator takes away any power the victim has to resist. Power is also removed when the abuse is discovered. Victims rarely tell about their abuse. The nature of image-based abuse makes it discoverable and available in the public domain. Therefore, victims have no control over how much they disclose or even the timing of disclosure. As previously discussed, victims also have no control over the distribution of their images. This lack of control can lead to a heightened sense of powerlessness in TACSA victims. It is therefore crucial that a sense of empowerment is conferred to victims of TACSA, to assist in their healing and recovery. Receiving support and advice from caring professionals appears to confer some degree of control and strength to victims which were eroded during the grooming stage and post-abuse. Victims speak of having someone stand with them and offering explanations and options allows them to explore their recovery and healing, including seeking justice against their offender. Many of the families MCF engages with share the common hope that one day their child will be able to move on, but the effects of trauma can often endure longer than expected. For a disproportionate number of children supported by MCF, schooling and friendship groups were disrupted with the ongoing fear of the images reemerging. This is usually coupled with therapeutic services either not being available due to waiting lists or practitioners wrongly fearing that their intervention may impede an investigation.

MCF’s experience with victims suggests that when presented with knowledge about the process of how TACSA occurs, or information about the post-discovery process with experienced and trauma-informed professionals, victims gained a renewed sense of control and hopefulness. This extended into positive conversations around future options for the victim. For one victim who was labeled disruptive by those in their education setting, learning about normative trauma responses was a significant milestone in their recovery. The family were no longer embarrassed and were able to apply strategies, rather than punish her for her completely appropriate post-abuse responses.

MCF is aware that our supportive actions can increase the confidence of parents and victims, reducing passivity to support that was offered to them. Most parents were acutely aware of how they presented to professionals and wanted to project a nice persona to help ingratiate themselves with practitioners in the hope that their positive interactions will increase the chance of their child receiving access to support. Despite MCF staff identifying and naming poor practice, this fear of no access to support often saw family members reluctant to formally complain, expressing concerns around any ramifications to their child if they did.

Many families described how having an advocate really helped, particularly in respect of access to a practitioner in MCF with a high level of knowledge around TACSA. When attending response meetings, families are often outnumbered within an environment familiar to safeguarding professionals and statutory partners, but who may not possess the most up-to-date awareness of current legislation or statutory guidance. Having a representative in the room helps alleviate any power imbalance. This can reduce parental and victim anxiety prior to meetings, ensuring meetings are more productive for all participants.

Professionals such as police, social workers, teachers and counselors are integral to empowering families to respond to their children’s needs. When these have limited knowledge of the subject, they are constrained in providing beneficial advice and guidance. Subject access requests carried out through support from MCF during direct work identifies a broad range of levels of interventions, with plans often not encapsulating the victim’s recovery needs. An MCF survey of practitioners identified that of the 167 participants only 9.5% expressed a high level of confidence when working with a child victim of TACSA. Therefore, it is important to identify how we can help the practitioner workforce improve their own confidence so that they can transfer a sense of empowerment to victims, supporting their recovery needs. Following support from MCF, one victim expressed the power of robust support in shoring up their own decision to reach out for support, stating

“…*I feel a bit proud of myself*…*I actually reached out. I’m glad I did. You believed me. I don’t feel alone.*”

## 10 Apportionment of blame

Despite ongoing progress in recent years in challenging victim blaming approaches to survivors of sexual abuse, it is still a significant factor faced by most victims and survivors. We see blame being applied from professionals, friendship groups, parents, and intimate partners. The relationship between the offender and victim is often conflated, resulting in a failure to appreciate the control of the abuser. This may lead to two detrimental outcomes, with the victim being either seen as the problem, and therefore not needing victim support or that their harm is deserved, as a consequence of their actions.

MCF has noticed through our direct work that children and their families are very attuned and sensitive to victim blaming statements. Furthermore, even when challenged some professionals try to defend these comments. This interplay appears to lean toward a subcategory of an “undeserving victim,” where the victim is harmfully labeled with blame for what happened to them. This may be underpinned by implicit and erroneous assumptions by professionals that the victim is one who should feel the shame of their actions and therefore are unworthy of comprehensive support. On an initial police visit, one parent expressed “that no one has ever seen their child as a victim,” with police records stating “I discussed the consequences to [the 12-year-old victim] of her actions in sending images to unknown people.” The school demonstrates further blame on the 12-year-old condemning her as “being a bit of a madam” when she was clearly in distress that the images of her abuse were being shared. This was a child showing clear signs of trauma, was self-harming and expressing suicidal thoughts after a sadistic offender had forced her to comply with their demands. Despite the clear mental health needs this child was never referred to mental health services by any professional.

Victims have frequently reported to MCF a sense of self-blame and responsibility for their abuse. This has included remarks that interrogate the victim’s response at the time, questioning why they did not challenge the offender. MCF direct response work often necessitates helping victims to understand where blame truly lies. Victims appear to be highly responsive to redirection of blame toward the offender(s) alone. In gently challenging self-blame, victims appear to experience a measure of freedom and relief that they were in no way responsible for the abusive conduct of the offending individual. Unpacking grooming methodology and taking time to help them understand why and how they were victimized is key to resituating blame on the offender, rather than the victim. This includes gentle challenge to any misconceptions within victims that they were “stupid,” rather than the offender being motivated and skillful in their offending behavior.

## 11 Normalization

Erikson (1965) looked at adolescence as a crucial period for the growth of a mature sense of self and personhood. For some of the children supported by MCF, their identity has become negatively formed through the abusive experience. This may an ascribed identity projected on to them by their peer group, if their abusive experience is widely known. One victim described being forced to send intimate recordings to her abuser. As part of her “punishment” the abuser identified her school and shared a number of these images with other children who attended there. The response was seen as sexual contact, rather than sexual abuse. The involved professionals expressed that she was “engaging in sexual behavior” and was widely seen by the school community as being the problem, rather than the offender who had sought her out. The victim persistently received sexual degrading comments for her peers and had virtually no friends. When a child at the school anonymously befriended her online and blackmailed her for more images, the victim expressed resignation and said he “sounded nice.” This demonstrates a form of negative normalization in victims regarding their abuse and their own sense of who they are because of it. It is important that this becomes healthily reconstructed within a safe and supportive therapeutic setting, allowing a victim to view themselves in a more positive light and worthy of normal, mutually fulfilling relationships.

An additional form of normalization which can be brokered within the professional response to abuse relates to education around how bodies react during abusive events. MCF engagement with victims post-TACSA has included victims expressing a common struggle with their physiological response to the abuse. Arousal and bodily responsiveness have left victims with significant discomfort and confusion over how their bodies could seemingly respond positively to sexual abuse. While this is something MCF routinely hears expressed, our practitioners have also noted how conversations which explore and educate victims on the normality of bodies reacting to sexual stimuli, even such that are harmful and abusive, can help victims to overcome their inner conflict over the disconnect between their bodily and mental responses to the abuse.

## 12 Discussion

Within a direct support environment with MCF, which is victim-centered and recovery-focused, victims and survivors are given space to express the mental toll flowing from what happened to them. This practitioner experience reveals the common mental health themes for victims-survivors of TACSA, which affect their wellbeing and recovery. Victims and survivors experience significant and complex mental health implications resulting from their TACSA experience. This is supported by extant research (Hanson, 2017a). Finkelhor et al. (2023) suggest that the permanency of abuse images in particular can intensify mental health impacts on victims.

It is apparent that there is some overlap between all the themes noted. Specifically, the presence of abuse documentation, (videos and/or pictures), and the lack of control over where that content was held or could be accessed, provoked extreme distress in victims-survivors. From this nexus, misplaced feelings of culpability (i.e., victim being to blame/at fault), complicity (i.e., victim assisting, not resisting) and responsibility (i.e., victim is accountable for their actions) co-mingled to compound the original TACSA experience. These three feelings were very closely related, but distinct, producing an overwhelming sense that they were capable of stopping the abuse and responsible for it in the first instance. Building on this, victims-survivors noted the chilling effect of the images of their abuse, effectively gagging them from any potential help-seeking. Victims either opted to be silent or felt forced to silence, believing that the evidence of their abuse may reinforce their feelings of culpability, complicity and responsibility. These consequences were also noted in Huber’s (2023) study, whose participant sample was women, but included some who had experienced image-based sexual abuse as children. Children experience specific vulnerability due to their stage of development and arguably these impacts are more acutely felt by children who may have less resilience to withstand the mental health consequences of their abusive experience.

Joleby et al. (2020) supports the notion of culpability, complicity and responsibility, with some victims recognizing the initial intrigue and flattery which undergirded the abusive experience, but which they have difficult in separating as offender tactics to abuse, rather than being an internal fault in the victim provoking the abuse. Schmidt et al. (2023) confirms the long-term mental health impacts of TACSA and the sense of guilt, shame and embarrassment that followed the abusive experience. This research also notes the sense of responsibility carried by victims for what happened to them, where they had not understood the manipulation and coercion which the offender had employed to secure their compliance.

Given the mental and emotional torment TACSA victims describe it is clear that clinical support services must be robust, with a fulsome appreciation of TACSA and its effects (Hamilton-Giachritsis et al., 2021). Schmidt et al. (2023) suggest that without a sound understanding of TACSA, victims can re-traumatization from counselors, through being misunderstood or inadvertently blamed for what happened to them. This can stall the victim from accessing better support elsewhere.

## 13 Conclusion

This practitioner report highlights the various expressions of mental and cognitive stress that victims-survivors of TACSA experience. As noted by Joleby et al. (2020), this can have proximate and distal reach on mental wellbeing. Furthermore, feelings of complicity and shame were also noted in this study. The technological medium was not only a tool to be abused by others but became a tool for psychological self-flagellation by victims who felt they should have been capable of just turning off the device and extracting themselves from the abusive scenario. Practitioner experience with victims of TACSA reveals the intense internal psychological processes that victims experience as they try to make meaning of their abusive experience. While physical symptoms may also be experienced, the heavy inner mental turmoil victims face indicates the profound and formative effect of such abuse. Successfully unpacking this during therapeutic sessions requires a rich understanding of the interplay of mental health impacts, with a need to actively dismantle unhealthy and incorrect understandings in TACSA victims about their abuse. Hanson (2017a) notes a variety of therapeutic interventions with good success post-TACSA, such as Cognitive Behavioral Therapy (CBT), Narrative Therapy and Eye Movement Desensitization Processing (EMDR). As noted by the author, these modalities can provide avenues for practitioners to clearly attribute blame solely on the offender, to normalize the victim’s reflexive physical and psychological responses to the abuse, allowing for a sense of empowerment to bloom in victims from a sound understanding of their TACSA experience. Such an approach can produce positive mental health effects for TACSA victims-survivors (Schmidt et al., 2023), predicated on a trusting relationship between practitioner and the victim-survivor (Quayle et al., 2023).

While TACSA and other CSA impacts may have comparable long-term impacts, the locus of the impact must be fully understood in order for the therapeutic needs to be properly and comprehensively addressed (Stänicke et al., 2024). Without proper training on the true understanding of the pervasive and enduring impact of TACSA on a victim’s wellbeing, clinical assistance may be shallow and ineffective (Schmidt et al., 2023). More research into these impacts and successful therapeutic treatment pathways is evidently warranted (Page et al., 2025), to illuminate and define the areas which cause victims mental distress and to reveal clinical approaches that alleviate this suffering.

## Data Availability

The datasets presented in this article are not readily available because of ethical, legal, and privacy-related concerns with sharing the data.
